# Mapping QTLs for *Pyricularia* leaf spot, nematode resistance, and yield related traits in pearl millet [*Cenchrus americanus* (L.) Morrone]

**DOI:** 10.3389/fpls.2025.1588485

**Published:** 2025-06-30

**Authors:** Sairam Vutla, Joseph E. Knoll, Anvesh Sankuratri, Risha G. Nayak, Limei Liu, Peng W. Chee, Raghupathy Karthikeyan, Bashasab Fakrudin, Mahendar Thudi, Patricia Timper, Karen Harris-Shultz, Jason G. Wallace, Hari Singh, Bharat Singh, Somashekhar M. Punnuri

**Affiliations:** ^1^ College of Agriculture, Family Sciences and Technology, 1005 State University Dr. Fort Valley State University, Fort Valley, GA, United States; ^2^ Crop Genetics and Breeding Research Unit, United States Department of Agriculture (USDA)-Agricultural Research Service, Tifton, GA, United States; ^3^ Institute of Plant Breeding, Genetics, and Genomics, University of Georgia, Tifton, GA, United States; ^4^ School of Plant and Environmental Sciences, Virginia Polytechnic Institute and State University, Blacksburg, VA, United States; ^5^ Department of Agricultural Sciences, College of Agriculture, Forestry and Life Sciences, Clemson University, Clemson, SC, United States; ^6^ University of Horticultural Sciences, Bagalkot, Karnataka, India; ^7^ Department of Crop & Soil Sciences, University of Georgia, Athens, GA, United States; ^8^ Center For Applied Genetic Technologies (CAGT), University of Georgia, Athens, GA, United States

**Keywords:** pearl millet, QTLs, physiological traits, nematode resistance, yield-related traits

## Abstract

Pearl millet [*Cenchrus americanus* (L.) Morrone, formerly *Pennisetum glaucum* (L.) R. Br.] is the sixth most important cereal globally and is used for forage and feed in the U.S. To identify genomic regions governing important physiological, agronomic and yield related traits, a recombinant inbred line population derived from the cross between Tift 99D_2_B_1 ×_ Tift 454 was phenotyped in the field in 2006, 2007 and 2013. In addition, the population was phenotyped for root-knot nematode resistance in the greenhouse during 2009. Using a previously generated genetic map containing 505 single nucleotide polymorphism markers and composite interval mapping, we identified 45 QTLs for eight traits (plant height, stem diameter, days to heading, panicle diameter, panicle length, 1000 seed weight, *Pyricularia* leaf spot disease, and root-knot nematode egg mass) across almost all linkage groups. These QTLs explained 6.31 to 32.51% of phenotypic variance for each trait and were consistently detected over different environments. Plant height and days to heading were colocalized on LG2 and LG5 showing maturity and plant height are linked and influence each other, similarly to other cereal crops. Interestingly, 5 of 19 QTLs linked to plant height, stem diameter, panicle diameter, and panicle length colocalized to the same locations on LG3, indicating breeding for one trait could simultaneously improve the other. The markers and genes identified in the present study can be used in developing high yielding pearl millet varieties using marker-assisted selection.

## Introduction

Pearl millet [*Cenchrus americanus* (L.) Morrone, formerly *Pennisetum glaucum* (L.) R. Br.; 2*n* = 2*x* = 14] is the sixth most important cereal globally, covering approximately 30 million hectares in Asia and Africa (Satyavathi et al., 2021). Globally, it serves as a staple food grain and fodder for over 90 million people (Srivastava et al., 2020; Satyavathi et al., 2021). It is grown in the United States, primarily for grazing, hay, cover crops, and wildlife. Pearl millet grain has a high nutritional value, characterized by high metabolizable energy, protein, micronutrient content and a low glycemic index (Agarwal et al., 2023). Nutritionally, the grain is gluten-free, making it suitable for specific dietary needs (Rai et al., 2008; Pei et al., 2022). On average, pearl millet contains approximately 12% protein, 69% carbohydrates, 5% lipids, 2.5% fiber, and 2.5% ash in terms of proximate nutritional composition (Andrews and Kumar, 1992; Tomar et al., 2021). Pearl millet has remarkable resilience to drought and heat stress, enabling cultivation in some of the most challenging environments (Gangashetty et al., 2016; Varshney et al., 2017). The above-ground portion of pearl millet is used as forage for dairy cows and other classes of livestock (Hancock et al., 2018). Pearl millet grain can also be used as feed for wildlife, poultry, beef cattle, and pigs (Andrews and Kumar, 1992). It has been grown in the southern U.S., particularly in Georgia, where there is potential for use by the poultry industry (Davis et al., 2003). However, there are few commercial grain hybrids available, (a notable exception is TifGrain 102), indicating a scope to develop hybrids suitable for this region (Hanna et al., 1991).

Pearl millet can grow on marginal soils with low fertility and organic matter content (Andrews and Kumar, 1992). Despite its positive attributes, pearl millet grain yields remain low, averaging around 0.85 tons/ha, which can be attributed to abiotic stresses (drought, low soil fertility) and biotic stresses such as nematodes, Striga (*Striga hermonthica*), millet head miner (*Heliocheilus albipunctella*), downy mildew (*Sclerospora graminicola*), leaf spot (*Pyricularia grisea*) and other diseases (FAOSTAT, 2020). By selecting and breeding pearl millet varieties with optimal panicle length, panicle diameter, and high 1000 seed weight, it is possible to enhance the resilience and productivity of this crop. These targeted improvements in yield components can lead to significant gains in grain yield, even under the challenging conditions posed by biotic and abiotic stresses (Poncet et al., 2000; Yadav et al., 2002, 2004; Vengadessan et al., 2013). Among biotic stresses in the southern region of the United States, plant-parasitic root-knot nematodes (RKN), *Meloidogyne incognita*, are important constraints in cropping systems (Timper and Wilson, 2006). Earlier efforts were made to identify root-knot nematode-resistant germplasm lines, and as a result, the inbred lines Tift 454 and Tift 99B were reported to be highly resistant and susceptible to RKN, respectively (Hanna et al., 2005a). In addition to root-knot nematodes, *Pyricularia* leaf spot (*Pyricularia grisea*) represents a yield limiting factor in pearl millet production (Timper et al., 2002). The commercial grain hybrid ‘TifGrain 102’ has been reported to exhibit strong resistance to *Pyricularia* leaf spot, providing a valuable resource for managing this disease (Hanna and Wells, 1989; Punnuri et al., 2016).

Understanding the underlying genetics and identifying the key genomic regions responsible for agronomic and yield-related traits will accelerate the development of climate-resilient varieties and enhance genetic gain (Varshney et al., 2018; Thudi et al., 2021; Yadav et al., 2021). Until recently, pearl millet was considered an orphan crop as it lagged behind sorghum (*Sorghum bicolor* (L.) Moench.) and other major cereals in terms of genetic improvement due to a lack of genomic resources (Passot et al., 2016). However, in recent years, genomic resources have dramatically expanded for this crop. High-density genetic maps for pearl millet were created using genotyping-by-sequencing (GBS) markers (Azhaguvel, 2001; Moumouni et al., 2015; Punnuri et al., 2016). Furthermore, significant efforts have been made to map the genomic regions responsible for abiotic stresses (Yadav et al., 2002; Sharma et al., 2011; Sehgal et al., 2012), and biotic stresses (Ambawat et al., 2016; Punnuri et al., 2016; Chelpuri et al., 2019). The genes involved in reducing internode length influencing plant height have been identified by map-based cloning and comparative mapping (Parvathaneni et al., 2013). In addition, with the availability of the pearl millet genome sequence and resequencing information of ~1000 germplasm lines, including wild species, pearl millet is now considered a genomics resource-rich crop (Varshney et al., 2017; Burgarella et al., 2018). These significant advances in genome sequencing in addition to high-density genetic maps will support genomics-assisted breeding (Punnuri et al., 2024).

There are very few studies that have mapped genomic regions for agronomic and yield-related traits using bi-parental populations arising from two dwarf, early maturing grain-type parents, particularly those used in producing a commercial hybrid. TifGrain 102 is a commercialized hybrid developed from Tift 99D_2_B_1_ and Tift 454, used in the current study, and has shown promising results in terms of its uniformity and yield stability. Therefore, mapping quantitative trait loci (QTLs) associated with yield and its components in this recombinant inbred line (RIL) population will provide greater insight into useful alleles and traits associated with these two parents. In this study, we report the QTLs for key agronomic and yield-related traits, as well as root-knot nematode resistance and *Pyricularia* leaf spot resistance, using previously generated GBS data (Punnuri et al., 2016) and phenotyping data generated during 2006, 2007, 2009, and 2013. In addition, we also report potential candidate genes in these QTL regions. The markers and genes reported in the present study can be used for genomics-assisted breeding of pearl millet for these important traits.

## Materials and methods

### Plant material

A RIL population comprising of 225 lines derived from the parental genotypes Tift 99D_2_B_1_ (female) and Tift 454 (Punnuri et al., 2016), was used for mapping QTLs for agronomic, yield related traits and biotic stress resistance. In brief, both parents are dwarf, early maturing grain-types that have been successfully used in producing a commercial hybrid known as TifGrain 102, where Tift 99B serves as the male-fertile maintainer line for the male-sterile Tift 99A (Hanna et al., 2005a, 2005b). Tift 454 is highly resistant to root-knot nematode (*Meloidogyne incognita*) (Hanna et al., 2005a), but it is susceptible to *Pyricularia* leaf spot. In contrast, Tift 99B is resistant to rust (*Puccinia substriata* var. *indica*) and other diseases, including leaf spot caused by *Pyricularia grisea*, but is vulnerable to root-knot nematode. Although both Tift 99B and Tift 454 differ only slightly in various traits, their differences fall within the typical range observed among cultivated grain-type pearl millet inbreds in the United States. Additionally, TifGrain 102 has shown strong resistance to *Pyricularia* leaf spot, making it a valuable resource for improving disease resistance in breeding programs (Hanna and Wells, 1989; Punnuri et al., 2016). F_6_ and F_7_ lines were used for phenotyping traits.

### Experimental design and trait phenotyping

A total of seven yield-related traits were phenotyped in the field over three years, though not all traits were assessed each year. In addition, the RIL population was also phenotyped for root-knot nematode resistance in the greenhouse in 2009 and for *Pyricularia* leaf spot disease incidence in 2013 (**Supplementary Table S1**). All experiments were arranged as randomized complete block designs. During 2006 and 2007 the F_6_ and F_7_ RIL populations, respectively were evaluated at Tifton (31.4505° N, 83.5085° W), Georgia, USA, with two replications each year. In 2013, 179-F_7_ RILs were evaluated at Old Farm (32.5344° N, 83.8961° W), Fort Valley State University (FVSU), Fort Valley, Georgia, with three replications.

In 2006, days to heading, grain yield (g/head), and plant height (cm) were collected from 225 F_6_ RILs, Tift 99D_2_B_1_, Tift 454, HGM100, and TifGrain102. Plant height was measured from the ground to the tip of the panicle. All three traits were measured on ten representative plants per plot and the average was taken. In 2007, the same population was planted as in the year 2006 but only 222 F_7_ lines were phenotyped due to poor germination and plant stand count (**Supplementary Table S2**). Phenotypic data collected in 2007 included grain yield (g/head), panicle diameter (mm), and panicle length (cm). In 2013, only 179 RILs from 184 RILs planted were phenotyped due to poor seed germination and plant losses caused by unfavorable field conditions. The traits evaluated in 2013 were plant height (cm), panicle length (cm), stem diameter (mm), panicle diameter (mm), days to heading, and seed weight for 1000 seeds (g). Plant height was measured from the base of the plant above ground till the flag leaf base (peduncle of the panicle). Panicle diameter and stem diameter were measured using digital Vernier calipers. Days to heading was counted from sowing till the complete emergence of panicle in 10 plants of each plot. Weight of 1000 seed was measured on one composite seed sample from each plot. Seeds were counted using a digital seed counter (Old Mill Equipment Co, USA).

### Evaluation for *Pyricularia* leaf spot disease

A F_7_ population (N=179) was grown in the field at Fort Valley (32°31’09.9”N 83°52’02.1”W) in 2013 in a randomized complete block design with three replications and was used to assess leaf spot disease. The same experimental block that was used for evaluating above traits in 2013 was also used for assessing *Pyricularia* leaf spot incidence. *Pyricularia* leaf spot infection occurred under natural conditions because of the rainy, humid weather during our experiment. Ten plants in each plot were visually scored and an average rating for the plot was used for analysis. The disease symptoms were very distinct on all RILs and the parental line (Punnuri et al., 2016). The disease scoring was performed as described by the International Crops Research Institute for the Semi-Arid Tropics (ICRISAT) using a 1–9 scale (Thakur et al., 2011), where 1 indicates no symptoms and 9 indicates complete plant death from disease. As disease progress is affected by growth stage, some of the late-maturing lines showed less severe symptoms. Therefore, disease scores for later-maturing RILs were adjusted based on their maturity and disease progress curve (Wilson and Hanna, 1992).

### Evaluation for nematode resistance

In 2009, a set of 180 RILs along with both inbred parents and their F_1_ hybrid were planted in 10 cm diameter pots in the greenhouse in a randomized complete block design with three replications. The susceptible parent, Tift 99D_2_B_1,_ was planted in three pots per replication, while the other lines were planted one pot per replication. Soil sterilization and greenhouse management was as described (Timper and Wilson, 2006). One vigorous plant per pot was retained after 10 days. *M. incognita* race 3, was cultured on eggplant (*Solanum melongena*) cv. Florida Market and 8000 eggs (4000 were added on the first day and another 4000 was added after two days) per pot were inoculated. Plants were removed from pots eight weeks after inoculation (two nematode generations) and examined for egg masses on roots. For this, roots were rinsed in water and stained in a 0.05% phloxine B solution for 3-5 minutes during which egg masses are stained bright red. Egg mass was estimated on a 0 – 5 scale (Holbrook et al., 1983) as follows: 0 = no egg mass, 1 = 1 – 2 egg masses, 2 = 3 – 10 egg masses, 3 = 11 – 30 egg masses, 4 = 31 – 100 egg masses, and 5 = greater than 100 egg masses. After rating the trials from first two replications, we observed that the egg masses were very small, so we waited two more weeks to let them grow in replication 3. The data recorded from each trial (1, 2 and 3) was averaged and used for analysis.

### Statistical analysis for field and greenhouse data

Mean values and ranges were calculated among RILs for all traits for the respective years. Correlations between traits were calculated with in each year using the CORR procedure of SAS v.9.4 (SAS Institute, Inc.).

The VARCOMP procedure of SAS was used to estimate variance components with the default method (MIVQUE0) and with RILs and replications as random factors. Variance components were used to calculate broad sense heritability (*H* ^2^) (Equation 1) using the following formula:


(1) $$H^{2}={\text{σ}}_{\text{g}}^{2}/({\text{σ}}_{\text{g}}^{2}\times {\text{σ}}_{\epsilon }^{2}/r)$$

where ${\text{σ}}_{\text{g}}^{2}$ is the variance due to genotype (RILs) and ${\text{σ}}_{\text{ϵ}}^{2}$ is the error variance and r is the number of replications.

The GLM procedure of SAS was used for the analysis of variance (Two-way ANOVA with a 5% level of significance) with genotype and replication as fixed effects. Fisher’s LSD test was done to determine differences among entries and replications with a 5% level of significance.

### G-model based single marker analysis

The genotype and phenotype dataset were also utilized for marker-trait analysis using the G-Model (Bernardo, 2013). SNP data were recoded as ‘1’, ‘-1’, ‘0’, and ‘0.5’ for the Tift 454 allele, the Tift 99D_2_B_1_ allele, a heterozygous allele, and missing data, respectively. The significance level for the marker effect was 0.00001 as recommended (Bernardo, 2013). RIL phenotype data that was not normally distributed was transformed by using log_10_ (x + 1).

### Genetic map construction and QTL analysis

The GBS-based SNP data generated by Punnuri et al. (2016) on the RIL population identified 1,191 core markers. To avoid problems created by segregation distortion, we excluded all markers with segregation distortion while constructing the genetic linkage map using a chi-square test with alpha of < 0.05 and hence used 505 non-distorted markers from the 1,191 core markers previously deployed. Each linkage group (LG) was grouped as per 7 chromosomes named in Punnuri et al. (2016). QTL analysis was carried out by composite interval mapping in Windows QTL Cartographer software v2.5_011 (Wang et al., 2012). For this, the SNP calls were encoded as ‘2’, ‘0’, and ‘-’ for the Tift 454 allele, the Tift 99D_2_B_1_ allele, and missing data, respectively. Missing phenotypes were represented by “period”. The LOD (log_10_ likelihood ratio) thresholds of significant QTLs were determined by performing 1000 permutations using a Type I error set at p < 0.001. The percentage of phenotypic variance explained by a QTL was calculated by multiplying the R² by 100. QTLs explaining more than 10% phenotypic variation explained (PVE) were considered major QTLs and < 10% phenotypic variation was considered as minor QTLs. The linkage map showing the identified QTLs was constructed using MapChart v2.32 (Voorrips, 2002). We chose to use a genetic map instead of a physical map in case the parental lines showed any significant deviations from the reference genome (insertions, deletions, inversions, etc.).

### Candidate gene analysis

The pearl millet genome sequence database available publicly (NCBI database) was used for gene identification. An in-house script was developed to identify candidate genes that resided near the identified QTLs. Genes that were within the QTL were identified and compiled for their primary functional annotation. Only genes with known functional annotations and Gene Ontology (GO) terms are reported.

## Results

### Performance of the population, variance components and broad-sense heritability

We identified significant variation among parents and RILs for most of the traits studied (**Table 1**). Tift 454 had higher average values than Tift 99B for all traits measured except stem diameter (2013), days to heading (2006), grain yield (2006), and nematode egg mass. For nematode egg mass, Tift 99B had a mean of 1.89 (out of 5), while Tift 454 had a mean of 0.0. For all agronomic traits, the F_1_ hybrid plants had greater values than either of its parents except for days to heading (2006 and 2013), *Pyricularia* leaf spot, and RKN egg mass. The mean values for most traits among the RILs were intermediate between the two parents. We observed significant variation in plant height between the 2006 and 2013 phenotypic evaluations due to differences in measurement. During the 2006 field evaluations at Tifton, plant height among RILs varied between 35-155 cm with an average height of 86.1 cm. In the 2013 field evaluations at Fort Valley, the height among RILs varied between 42.5-109 cm with an average height of 73.4 cm. The broad-sense heritability of the plant height trait at both locations was high (H^2^ = 0.877 in 2016 and H^2^ = 0.756 in 2013). The stem diameter measured in 2013 varied between 1.50 to 6.74 mm with an average of 4.29 mm and had a heritability of 0.837 (**Table 1**). During 2013, days to heading varied between 42.7 to 57.7 days with an average of 47 days, while days to heading at Tifton in 2006 varied between 39.1 to 59.5 days with an average of 45.6 days. Nevertheless, heritability for days to heading was slightly higher in 2006 (H^2^ = 0.853). We observed heritability of 0.809 and 0.852 during 2013 and 2007, respectively, for panicle diameter. During 2013, 1000 seed weight for RILs varied between 5.9 to 12.2 g with an average weight of 8.7 g and heritability of 0.829. Grain yield per panicle varied between 0.14 to 15.3 g with an average of 3.08 g during 2007, while in 2006, grain yield varied between 0.07 to 12.5 g per head with an average of 3.83 g. The heritability for grain yield was 0.789 and 0.824 during 2007 and 2006, respectively (**Table 1**). The egg mass rating values of RILs showed a range of 0 to 3.33 with a mean of 1.37 for all trials and heritability of 0.718.

**Table 1:** Summary of phenotypic variation among parents, F_1_ and RILs. Mean values, range, standard deviation and heritability values are for the RILs.

Trait	Year	Tift99B	Tift454	F_1_	RILs				
		Mean	Mean	Mean	Minimum	Maximum	Mean	St. Dev.	H^2^
Plant Height (cm)	2006	70.0	110	125	35.0	155	86.1	16.0	0.877
	2013	63.2	80.5	108	42.5	109	73.4	12.3	0.756
Stem Diameter (mm)	2013	4.41	3.58	6.61	1.50	6.74	4.29	1.14	0.837
Days To Heading (d)	2006	45.1	43.2	44.6	39.1	59.5	45.6	4.77	0.853
	2013	44.0	48.3	47.0	42.7	57.7	47.0	3.31	0.766
Panicle Length (cm)	2007	18.5	26.8	29.8	14.6	30.4	22.2	3.29	0.859
	2013	13.3	21.8	26.2	11.5	26.7	17.6	3.20	0.678
Panicle Diameter (mm)	2007	23.5	24.4	27.5	16.7	31.4	22.2	2.69	0.852
	2013	12.7	15.8	18.0	2.76	25.2	14.4	3.62	0.809
Grain Yield (g/head)	2006	5.06	4.82	7.09	0.07	12.5	3.83	2.26	0.824
	2007	2.88	5.96	9.53	0.14	15.3	3.08	2.14	0.789
1000 Seed Weight (g)	2013	8.83	10.33	12.7	5.9	12.2	8.7	0.9	0.829
*Pyricularia* Leaf Spot (1-9)	2013	3.0	7.3	2.0	1.0	9.0	5.6	1.8	0.939
Egg mass Avg_trial (1,2,3)	2009	1.89	0.0	1.33	0.0	3.33	1.37	1.05	0.718
Egg mass trial (1&2)	2009	1.66	0.0	1.50	0.0	3.50	1.25	1.15	0.731
Egg mass trial 3	2009	2.33	0.0	1.00	0.0	4.0	1.60	1.40	–

H^2^, Broad-sense heritability; St. Dev., Standard Deviation.

### Genetic correlations between different traits

Correlations between traits were observed within each year (**Supplementary Table S3**). The highest correlations in 2013 were observed between stem diameter and panicle diameter (0.59), plant height and stem diameter (0.49), and plant height and panicle length (0.56). In 2007, moderate correlations were observed between panicle length and panicle diameter (0.42), as well as grain yield per head and panicle diameter (0.40). In 2006, all traits measured were weakly correlated.

### G-model results

Single marker analysis using the G model revealed several markers associated with the different traits measured in the pearl millet mapping population (**Table 2**). There were five markers directly associated with five different traits on four linkage groups. SNP marker S3_2921 was associated with plant height (2013), panicle length (2013), and panicle diameter (2007). SNP marker S3_2737 was associated with panicle length (2007), panicle diameter (2013), and stem diameter (2013). The markers associated with panicle length and panicle diameter for 2007 and 2013 and plant height (2013) on linkage group 3 were in an overlapping region.

**Table 2:** Single nucleotide polymorphism markers identified as being associated with pearl millet traits using the G-Model.

Trait	Year	Associated SNP	LG	Position (cM)	Marker effect	p-value
Plant Height	2006	S2_6957	2	39.53	0.63	9x10^-7^
	2013	S3_2921	3	14.79	4.27	5x10^-7^
Stem Diameter	2013	S3_2737	3	14.36	0.4	7.8x10^-6^
Days To Heading	2006	S5_0677	5	3.6	-0.02	8x10^-7^
Panicle Diameter	2013	S3_2737	3	14.36	1.76	0
	2007	S3_2921	3	14.79	1.49	0
Panicle Length	2007	S7_0716	7	14.15	-0.81	3.5x10^-6^
	2007	S3_2737	3	14.36	1.63	0
	2013	S3_2921	3	14.79	1.48	0

### Genetic map construction and QTLs mapping

**Figure 1:**
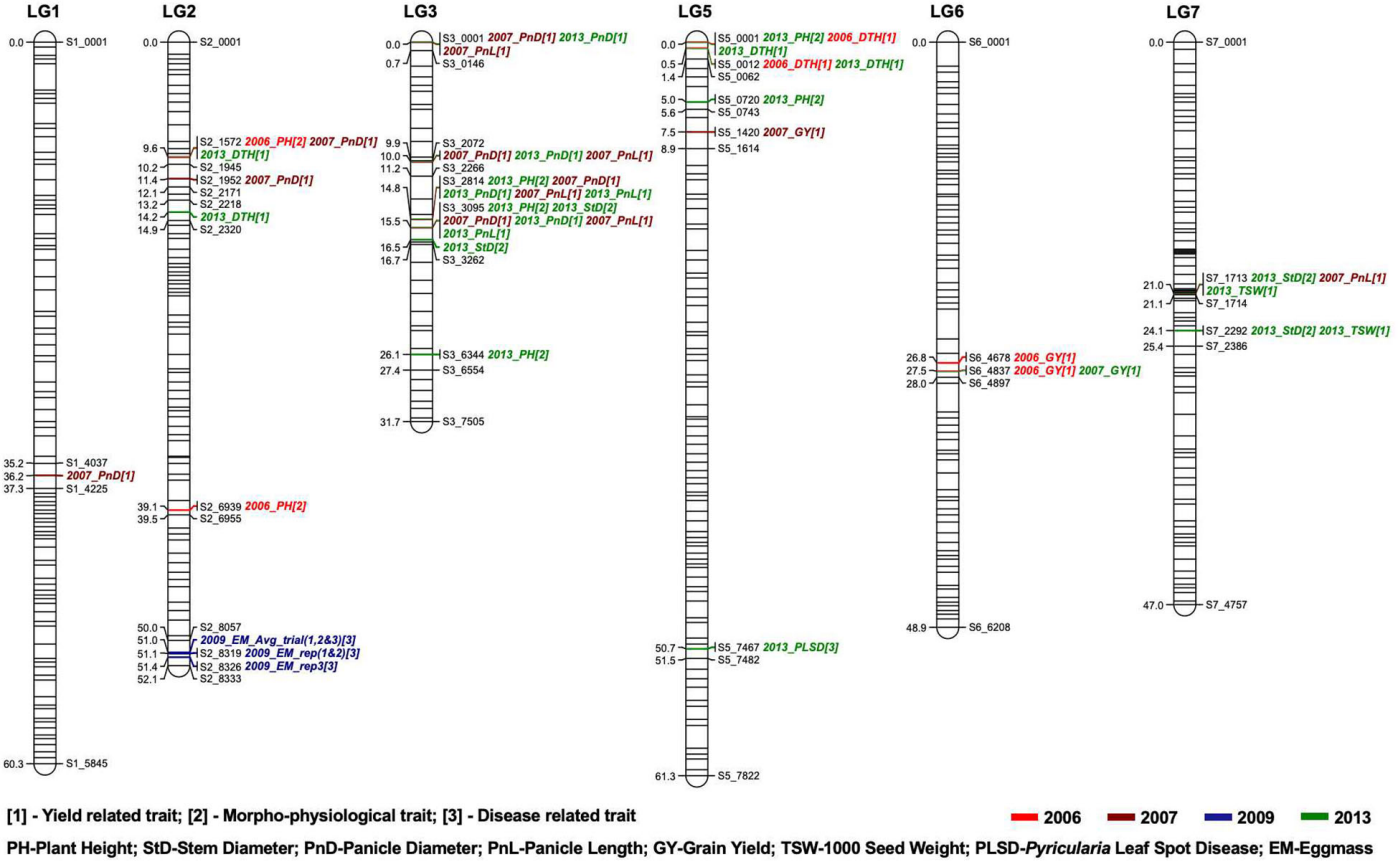
Genetic map constructed using RIL populations derived from Tift 99D_2_B_1_ × Tift 454 comprise 505 SNP markers. Number of markers mapped ranges from 35 to 89 per linkage group. Linkage distances are described with a scale in cM on the left side and the QTLs regions are given on the right side of each bar.

A total of 505 non-distorted markers were mapped on the seven linkage groups of pearl millet. The number of SNP markers mapped per linkage group (LG) ranged from 35 to 89 with an average of 72.14 markers per linkage group (**Supplementary Figure S1**; **Supplementary Table S4**). LG3 had the most markers (89 SNPs), followed by LG7 (85 SNPs) and LG5 (82 SNPs). Exclusion of segregation distortion markers reduced our original map to very few markers on all linkage groups. Regions such as LG3 and LG4 were disproportionately affected and LG4 also had less polymorphic markers to begin with due to shared ancestry. We identified QTLs on all chromosomes (LGs) except chromosome 4 for all traits studied across different years: plant height (2006 & 2013) on LG2, LG3, and LG5; stem diameter (2013) on LG3 and LG7; days to heading (2006 & 2013) on LG2 and LG5; panicle diameter (2007 & 2013) on LG1, LG2 and LG3; panicle length (2007 & 2013) on LG3 and LG7; grain yield (2006 & 2007) on LG5 and LG6; 1000 seed weight (2013) on LG7; *Pyricularia* leaf spot disease (2013) on LG5; and nematode egg mass (2009) on LG2 (**Table 3**). In total, we identified 45 QTLs on six linkage groups using composite interval mapping with LOD> 3.00 (**Figure 1**).

**Table 3:** Summary of QTLs identified for morphological and yield-related traits based on phenotype of the pearl millet RIL population (Tift99B × Tift454) during 2006, 2007, 2009 and 2013.

Trait	Year of phenotyping	Linkage group	Position (cM)	Left marker	Right marker	LOD	PVE (%)	Additive
Plant Height (cm)	2006	2	9.64	S2_1572	S2_1945	3.07	6.38	-0.03
	2006	2	39.12	S2_6939	S2_6955	3.06	6.70	-0.03
	2013	5	0.01	S5_0001	S5_0012	3.54	7.20	-0.02
	2013	5	4.97	S5_0720	S5_0743	5.11	10.16	-0.02
	2013	3	14.8	S3_2814	S3_3095	4.69	9.58	-0.02
	2013	3	15.46	S3_3095	S3_3262	4.89	10.02	-0.03
	2013	3	26.14	S3_6344	S3_6554	3.91	8.27	-0.02
Stem Diameter (mm)	2013	3	15.46	S3_3095	S3_3262	4.68	9.83	-0.05
	2013	3	16.46	S3_3095	S3_3262	5.04	10.59	-0.05
	2013	7	24.1	S7_2292	S7_2386	5.99	13.25	0.06
	2013	7	21.03	S7_1713	S7_1714	7.74	16.99	0.07
Days To Heading (d)	2006	5	0.01	S5_0001	S5_0012	6.43	13.43	0.02
	2006	5	0.48	S5_0012	S5_0062	6.86	14.22	0.02
	2013	5	0.01	S5_0001	S5_0012	4.93	10.68	0.01
	2013	5	0.48	S5_0012	S5_0062	4.76	10.34	0.01
	2013	2	9.64	S2_1572	S2_1945	3.74	8.38	-0.01
	2013	2	14.17	S2_2218	S2_2320	5.34	11.98	-0.01
Panicle Diameter (mm)	2007	1	36.19	S1_4037	S1_4225	4.55	8.09	0.02
	2007	2	9.64	S2_1572	S2_1945	3.76	6.29	-0.01
	2007	2	11.42	S2_1952	S2_2171	3.92	6.54	-0.01
	2007	3	0.01	S3_0001	S3_0146	4.60	9.69	-0.02
	2007	3	9.95	S3_2072	S3_2266	8.87	17.99	-0.02
	2007	3	14.8	S3_2814	S3_3095	11.10	21.60	-0.03
	2007	3	15.46	S3_3095	S3_3262	10.44	20.71	-0.03
	2013	3	0.01	S3_0001	S3_0146	3.24	6.29	-0.03
	2013	3	9.95	S3_2072	S3_2266	5.88	10.56	-0.05
	2013	3	14.8	S3_2814	S3_3095	6.12	10.79	-0.05
	2013	3	15.46	S3_3095	S3_3262	5.84	10.41	-0.05
Panicle Length (cm)	2007	7	21.03	S7_1713	S7_1714	3.03	6.02	0.02
	2007	3	0.01	S3_0001	S3_0146	3.56	6.77	-0.02
	2007	3	9.95	S3_2072	S3_2266	11.58	27.00	-0.04
	2007	3	14.8	S3_2814	S3_3095	8.97	22.78	-0.03
	2007	3	15.46	S3_3095	S3_3262	8.02	20.43	-0.03
	2013	3	14.8	S3_2814	S3_3095	4.97	10.20	-0.03
	2013	3	15.46	S3_3095	S3_3262	4.89	10.14	-0.03
Grain Yield (g/head)	2006	6	26.77	S6_4678	S6_4837	5.95	16.02	0.13
	2006	6	27.52	S6_4837	S6_4897	5.07	13.47	0.12
	2007	6	27.52	S6_4837	S6_4897	3.45	7.08	0.10
	2007	5	7.47	S5_1420	S5_1614	4.89	10.22	-0.10
1000 Seed Weight (g)	2013	7	21.03	S7_1713	S7_1714	4.09	10.52	0.02
	2013	7	24.1	S7_2292	S7_2386	4.56	11.50	0.02
*Pyricularia* Leaf Spot	2013	5	50.7	S5_7467	S5_7482	3.32	9.23	-0.58
Egg mass Avg trial (1,2&3)	2009	2	50.99	S2_8057	S2_8319	10.72	28.37	1.12
Egg mass trial (1&2)	2009	2	51.06	S2_8319	S2_8326	11.78	32.51	0.13
Egg mass trial 3	2009	2	51.42	S2_8326	S2_8333	8.94	23.84	0.14

### QTLs for morpho-physiological traits: plant height and stem diameter

We identified a total of seven QTLs for plant height on linkage groups (LG) 2, 3 and 5 (**Table 3**). The phenotypic variance explained by these QTLs ranged from 6.38-10.16%. Further, among these QTLs, the QTL based on 2013 phenotyping data on LG5 explained the maximum phenotypic variation, 10.16% (**Table 3**). Nevertheless, all seven QTLs had a negative additive effect on the trait, meaning that increased height is associated with the Tift 454 allele. In the case of stem diameter, we identified four QTLs with two each on LG3 and LG7. Interestingly the QTLs for plant height and stem diameter are co-localized at 15.46 cM on LG3 flanked by the two SNP markers S3_3095 and S3_3262. Like plant height, the QTL identified for stem diameter also had the negative additive effect on the trait.

### QTLs for yield related traits- days to heading, panicle diameter, panicle length, grain yield, 1000 seed weight

Days to heading, panicle diameter, panicle length, grain yield per head and 1000 seed weight are the key yield related traits that directly impact grain yield in pearl millet. We identified six QTLs for days to heading and 11 QTLs for panicle diameter and seven QTLs for panicle length, four QTLs for grain yield and two QTLs for 1000 seed weight (**Table 3**). Further, among these QTLs several identified were overlapping and consistent across different years. For example, a QTL for days to heading on LG5 at 0.48 cM was identified in 2006 and 2013, demonstrating the consistency of this trait across years. No QTLs were detected for days to heading based on 2007 phenotypic evaluations of the RIL population. The QTLs for days to heading explained 8.38 to 14.22% of the phenotypic variation for the trait. In case of panicle diameter, we identified seven QTLs in 2007 on LG1, 2 and 3, of which four detected on LG3 were consistent with QTLs detected in 2013. The phenotypic variation explained for panicle diameter ranged from 6.28 to 21.60%. In the case of panicle length, seven QTLs were detected on LG3 and LG7 for 2007 and 2013 evaluations. Panicle diameter and panicle length shared four common QTLs on LG3 over the years. Further among these eight QTLs detected for panicle length and panicle diameter, four QTLs were based on 2007 evaluations and four based on 2013 (**Figure 1**). However, no QTLs were identified based on 2006 evaluation data for both the traits. For panicle length, five QTLs were detected in 2007 on LG3 and LG7, of which two overlapped with the 2013 evaluations and the PVE ranged from 6.01 to 26.99%. There were five genomic positions on LG2 (9.64 cM) for days to heading and panicle diameter and LG3 (0.01 cM, 9.95 cM, 14.8 cM, 15.46 cM) for panicle diameter and panicle length that were consistently identified in 2007 and 2013 with the same marker interval having the highest LOD and PVE. Furthermore, most of the QTLs for both panicle length and diameter had negative additive effects on the traits, meaning that greater panicle length and diameter were associated with Tift 454 alleles. In the case of grain yield, four QTLs were detected, of which two (at 27.52 cM) overlapped for 2006 and 2007. Two QTLs were detected for 1000 seed weight in 2013, of which one overlapped with panicle length and stem diameter on LG7 at 21.03 cM (**Table 3**).

### QTLs for *Pyricularia* leaf spot disease

We identified one QTL on LG5 at 50.7 cM between S5_7467 and S5 7482 with 9.23% PVE and a negative additive effect of 0.58. Since a higher number indicates greater disease, resistance is associated with the Tift 99B allele.

### QTLs for nematode resistance

We evaluated the RIL population for root-knot nematode resistance by rating egg masses in greenhouse experiments in 2009. All three replicates were analyzed, as well as replicate 1 and 2, or just replicate 3 due to difference in phenotyping of replicate 3 as compared to 1 and 2. Three QTLs on LG2 explained 23.84-32.51% of phenotypic variation were identified (**Table 3**, **Figure 1**). Notably, QTLs identified in the present study are the major QTLs located between S2_8057 and S2_8333, and have positive additive effects on the trait, meaning that resistance is associated with the Tift 454 allele.

### Candidate genes for different traits

A total of 19,762 genes were identified between the markers flanking 45 QTL regions identified in this study. Of these, 13,214 genes are identified in the major QTL regions (**Supplementary Table S5**). And also among these, 6884 are unique genes in these QTL regions (**Supplementary Tables S6**, **S7**). A large number of unique genes were identified in the grain yield QTL region (4091 genes). A set of 294, 408, 868, 1045, 1124 and 1526 unique genes were identified for stem diameter, RKN egg mass, panicle length, days to heading, panicle diameter and plant height respectively. We identified only one gene for *Pyricularia* leaf spot. Interestingly all 127 and 1526 genes identified in the 1000 seed weight and plant height QTL regions are unique. The known relevant genes for these traits were extracted with Gene Ontology (GO) terms. The genes Pgl_GLEAN_10004502 and Pgl_GLEAN_10018466, that encode a heat shock protein Hsp90 are present in the QTL regions of days to heading (**Supplementary Tables S6**, **S7**). Among these 19,762 genes identified in the QTL regions, we summarized the number of genes in each QTL region for a given trait. Further, on functional annotation, 9057 genes had no reported function, and the remaining 10,705 genes encode for known functional proteins. We have focused discussion only on those genes that are reported to have influenced the trait in model species or known crop plants or that could be deployed in breeding programs for pearl millet improvement. These genes are also prioritized based on existing literature and previously characterized genes.

## Discussion

Pearl millet is a resilient, drought-tolerant crop that flourishes in harsh environments. Historically labeled as an orphan crop due to its limited genomic resources, recent advancements in next-generation sequencing (NGS) technologies have revolutionized genomic research in pearl millet. Previously, we developed a comprehensive set of 16,650 markers, including 1,191 core markers, which facilitated the detection of numerous quantitative trait loci (QTLs) that might have been missed in less saturated maps (Punnuri et al., 2016). In this study, we constructed a genetic map using 505 SNP markers derived from the core markers, excluding those with segregation distortion, to identify QTLs associated with morphological, disease resistance and yield-related traits in a RIL populations at two distinct locations.

The parental inbreds used for developing RILs in this study are Tift 454 and Tift 99B. Tift 454 and Tift 99A are the parents of TifGrain 102, with Tift 99B serving as a male fertile maintainer line for Tift 99A (Hanna et al., 2005a, 2005b). The parental line Tift 454 exhibits high resistance to the root-knot nematode species *Meloidogyne incognita* (Kofoid & White) Chitwood (Hanna et al., 2005a); Tift 99B is resistant to rust (*Puccinia substriata* var. *indica*) and other diseases such as leaf spot caused by *Pyricularia grisea*. Although the differences between the two parents are minimal for several traits, they fall within the expected ranges for cultivated grain-type pearl millet inbreds in the United States.

Our study revealed significant variation among the RILs for all traits examined. Traits with the highest heritability values (0.78-0.93) included 1000 seed weight (2013), grain yield (2006, 2007), panicle diameter (2007, 2013), stem diameter (2013), and *Pyricularia* leaf spot disease (2013). Similarly, high broad-sense heritability values ranging from 0.52 (panicle length) to 0.86 (plant height) were reported for panicle diameter and plant height (Kumar et al., 2021). Selecting traits with higher heritability is a powerful approach in genetics and breeding, as it leverages the genetic component of trait variation for more effective and predictable improvements.

In this study, we utilized GBS-based SNP markers to construct a high-density genetic map and identify QTL. Traditionally, QTL analyses in pearl millet have predominantly used SSR markers or DArT markers (Ambawat et al., 2016; Kumar et al., 2017, 2021). However, a few studies have employed GBS-based SNPs (Punnuri et al., 2016; Pucher et al., 2018) or SSCP-SNP markers (Bertin et al., 2005). Most of SSR-based genetic maps contained very few markers, for instance, a consensus map combining four different populations had only 174 loci (Rajaram et al., 2013). GBS-based maps often have several hundred markers (314 for Moumouni et al., 2015; 460 for Pucher et al., 2018; and 505 for the current study). High-density genetic maps enable identification of precise genomic regions for use in breeding programs for trait improvement (Punnuri et al., 2024).

All the traits were not measured across all years and locations, but only four traits are common across two seasons and locations. Although a prior study showed that eight QTLs explained 42.7% of observed phenotypic variation for panicle length and five QTLs explained 45.8% of observed phenotypic variation (Vengadessan et al., 2013), our research identified 29 major QTLs (explaining >10% of PVE each) for traits such as plant height, stem diameter, days to heading, panicle diameter, panicle length, grain yield, 1000 seed weight, leaf spot resistance, and root-knot nematode egg masses. Notably, the total PVE for egg mass trial (1&2), egg mass average trial (1,2&3) and egg mass trial 3 were 32.51%, 28.37%, and 23.84%, respectively. Additionally, we identified 16 minor QTLs (explaining <10% PVE each) across all traits.

These QTLs were detected with high LOD values (ranging from 3.02 to 11.78) on all linkage groups (LGs) except LG4, explaining significant phenotypic variation for yield-related traits, including biotic resistance. Most of these QTLs were consistently expressed across different locations, years, and traits. LG4 showed less polymorphic markers associated with QTLs due to shared ancestry between the parents (Punnuri et al., 2016).

The highest number of QTLs (19) were detected on LG3, showing overlap among traits and years. For instance, plant height (2013), panicle diameter (2007 & 2013), and panicle length (2007 & 2013) were consistently observed at 14.8 cM on LG3 across different experimental locations. Similarly, plant height (2013), stem diameter (2013), panicle diameter (2007 & 2013), and panicle length (2007 & 2013) were identified on LG3 at 15.46 cM between markers S3_3095 and S3_3262. Panicle diameter (2007 & 2013) and panicle length (2007) were consistently found on LG3 at 0.01 cM and 9.95 cM between evaluations in Tifton and Fort Valley. Among these four traits (plant height, stem diameter, panicle length and panicle diameter), QTLs for two traits (panicle length and panicle diameter) had four overlapping QTL that were consistently identified at 0.01 cM, 9.95 cM, 14.8 cM, and 15.46 cM on LG3. These co-localized QTL could represent pleiotropic effects or tightly linked genes. Determining which of these is actually the case will require the development of fine-mapping populations to further break up the linkage blocks in this region, and may benefit from multi-trait QTL analyses.

Other notable QTLs included plant height (2006), days to heading (2013), and panicle diameter (2013) at 9.64 cM on LG2. LG2 also harbored the nematode resistance QTL between markers S2_8057 and S2_8333. Leaf spot caused by *Pyricularia* due to frequent rains and high humidity is a major concern in the southeastern United States. The QTLs reported for leaf spot are different from our previous report (Punnuri et al., 2016) as we removed distorted markers and used composite interval mapping in the current study. Nevertheless, we report identification of QTL for *Pyricularia* leaf spot in this study confirming the presence of a genomic region governing *Pyricularia* leaf spot on LG5. Earlier one RAPD marker (OP-D11700, 5.6 cM) was reported to be linked to *Pyricularia* leaf spot resistance using a population derived from Tift85DB and Tift65 (Morgan et al., 1998).

Grain yield-related QTLs (2006 & 2007) were found on LG6 in close proximity with overlapping regions at 27.52 cM. Similar results were found for grain yield on LG6 in a study by Kumar et al. (2017). Panicle length (2007) was linked with 1000 seed weight and stem diameter on LG7. These results indicate that most yield and domestication-related traits, such as panicle morphology and disease resistance, were co-localized in clusters on specific genomic regions. These regions were likely selected as linkage blocks during domestication for yield improvement. Consequently, many traits remained undetected on LG4. This is likely due to both biological and technical reasons. Biologically, the parental lines Tift 454 and Tift 99B share a common ancestry and both possess the *dw2* dwarfing gene derived from Tift 23D_2_ (Punnuri et al., 2016), which resulted in reduced polymorphism on LG4. Technically, LG4 had fewer markers compared to most other linkage groups, which limited our ability to detect QTLs due to reduced resolution and statistical power.

Previous studies using multi-location phenotyping data on a RIL population derived from ICMB 841-P3 × 863B-P2 identified 13 QTLs for plant height, 11 QTLs for panicle length, and 14 QTLs for 1,000-seed weight (Kumar et al., 2017). Vengadessan et al. (2013) reported QTLs for panicle length and diameter on LG3. Additionally, for panicle length, five major QTLs were mapped on LG3 in the current study, which is consistent with the findings of Vengadessan et al. (2013). A minor QTL for panicle length was also identified on LG7 in this study, supporting previous reports by Vengadessan et al. (2013) and Kannan et al. (2014). Recently, Gupta et al. (2024) identified 34 Meta-QTL regions with a 3.63-fold reduction in confidence intervals, harboring genes for agronomic and yield-related traits (plant height, flowering time) and yield-related traits (panicle length, 1000 grain weight, seed yield per plant). In a genomic scanning and genome-wide association studies, 314 SNPs detected on chromosomes 1, 2 and 5 were found to be associated with flowering and agromorphologic traits, of which the majority of them were linked with panicle length and width (Faye et al., 2022). Also, it was shown that presence of the alternate allele on SNP Chr3_at 18,486,380 bp, increased 1000 seed weight. In other studies, major and stable QTL associated with flowering, number of productive tillers, ear head length, and test weight were mapped on chromosomes 1 and 3 using a RIL population derived from a cross between PT6029 and PT6129 (Subbulakshmi et al., 2024). Similar to Kannan et al. (2014), in the current study a QTL for days to heading was mapped on LG5 for 2006 and 2013 phenotyping years.

In this study we reported consistent and stable QTLs for panicle diameter and panicle length that shared the same genomic regions on LG3; however, the meta-QTL analysis performed by Gupta et al. (2024) based on 12 independent studies for various traits harbored QTLs and genes for plant height, 1000 seed weight, panicle length, seed yield per plant, and flowering time in the Meta-QTL1.1 on LG1. Nevertheless, QTLs for panicle length and panicle diameter were present in Meta-QTL3.6 on LG3. The co-localized QTLs reported in our study can be used in marker assisted introgression for simultaneous improvement of several traits. Grain number and grain mass are critical yield components that directly influence grain yield (Kumar et al., 2017). Panicle length significantly affects panicle weight, which is a key factor in calculating the panicle harvest index. QTLs for mean grain yield across various moisture environments have been mapped to linkage groups LG2, LG3, and LG4 under different post-flowering moisture conditions (Bidinger et al., 2007). In the current study, QTLs for plant height, panicle length, panicle diameter, and stem diameter were localized on LG3, supporting previous findings. A QTL for 1000 seed weight on LG7 was previously reported in two different mapping populations (Kumar et al., 2021, 2017), similar to our findings but at different positions on LG7.

A recent study using a RIL population derived from ICMS 8511-S1-17-2-1-1-B-P03 × AIMP 92901-S1-183-2-2-B-08 identified 15 QTLs for plant height scattered across seven chromosomes, five QTLs for panicle traits dispersed across four chromosomes, and two QTLs for thousand grain weight spanning LG3 and LG7 (Kumar et al., 2021). The co-localization of QTLs offers significant advantages in pearl millet breeding by enabling the simultaneous improvement of multiple traits or leveraging the selection of one trait to enhance others. In this study, we also identified stable QTLs for traits such as days to heading, panicle diameter, and panicle length that were consistently mapped to the same positions across different years. These stable QTLs can be utilized in marker-assisted selection to improve these traits cost-effectively.

Several studies have identified key yield-related genes in pearl millet, shedding light on their chromosomal positions and functional roles. Four genes, (Pgl_GLEAN_10031557, Pgl_GLEAN_10008690, Pgl_GLEAN_10007548 and Pgl_GLEAN_10005530) identified in this study encoding phospholipase were identified in the panicle diameter and grain yield QTL regions are believed to be responsible for determining pearl millet yield. The results from earlier studies further confirm the role of phospholipase gene on grain yield of pearl millet. For instance, a genome-wide analysis of the phospholipase gene family revealed 44 genes distributed across the seven chromosomes, with the highest concentration on chromosome 1 (12 genes), followed by chromosome 5 (8 genes), chromosome 6 (7 genes), and fewer on the remaining chromosomes. These phospholipase genes are implicated in various physiological processes, influencing stress responses that ultimately affect yield (Moin et al., 2024). Flowering time, a crucial determinant of yield in pearl millet, is regulated by several genes located primarily on chromosome 2. Among them, *PhyC* (PHYTOCHROME C) plays a pivotal role in the photoperiodic induction of flowering, with polymorphisms near this gene linked to variations in flowering time and yield components. Similarly, *FRS12* (FAR1-RELATED SEQUENCE 12) and *Hd3a* (HEADING DATE 3a) have been identified as key regulators of the transition from vegetative to reproductive stages. We identified a gene Pgl_GLEAN_10007711 in the panicle diameter QTL that encodes for FAR1. Additionally, *OsHAC1* (HISTONE ACETYLTRANSFERASE 1), also found on chromosome 2, is involved in chromatin remodeling, affecting gene expression related to flowering (Faye et al., 2022). We identified the gene Pgl_GLEAN_10010455 in grain yield QTL region encodes for histone acetyltransferase. Beyond flowering regulation, drought tolerance is another critical factor influencing yield stability in pearl millet. A comprehensive study identified 74 drought-associated genes distributed across all seven chromosomes, with chromosome 1 containing the highest number (14 genes), followed by chromosomes 6 (12 genes), 4 (11 genes), and others with fewer genes. These drought-responsive genes function in various pathways that enhance resilience under water-limited conditions, contributing to yield improvement under abiotic stress (Chakraborty et al., 2022).

In addition, genes that are indirectly related to yield were also reported, for instance, *PmAAP1* (Amino Acid Permease 1) and *PmGS1* (Glutamine Synthetase 1) play crucial roles in nitrogen assimilation and amino acid transport, which indirectly contribute to yield by improving grain nutritional quality (Singh et al., 2024b). In our study, the gene Pgl_GLEAN_10003392 present on chromosome 7 encodes for glutamine synthetase and is present in the grain yield QTL region. Similarly, the gene Pgl_GLEAN_10022286 present on chromosome 7 in the grain yield QTL region encodes for amino acid permease. Furthermore, resistance to foliar blast, a major disease affecting pearl millet yield, has been linked to several genes identified in a GWAS of 281 inbred lines (Singh et al., 2024a). Among them, *PmNPR1* (Nonexpressor of Pathogenesis-Related Genes 1) and *PmWRKY45* (WRKY Transcription Factor 45) were found to be key regulators of blast resistance, mainly located on chromosomes 1, 2, and 6. These genes enhance disease resistance by modulating defense pathways, ultimately stabilizing yield under biotic stress (Singh et al., 2024b). In contrast, in this study, we identified Pgl_GLEAN_10014680 on chromosome 7 within a grain yield QTL region, which is annotated as a pathogenesis-related gene. While pathogenicity is a trait typically associated with pathogens, host plants also harbour genes that influence disease outcomes, either by enhancing susceptibility or activating defence responses. The presence of Pgl_GLEAN_10014680 in a yield-associated QTL suggests a potential link between disease response and productivity, warranting further functional validation to determine its role in host-pathogen interactions. Additionally, rhizosheath formation, which influences water and nutrient uptake in pearl millet, has been linked to *PmEXPA1* (Expansin A1) and *PmMATE37* (Multidrug And Toxic Compound Extrusion 37). In this study, we identified the gene Pgl_GLEAN_10006620 in the grain yield QTL region that encodes for expansin. These genes regulate root-soil interactions and help plants maintain productivity in arid conditions, indirectly enhancing yield stability in drought-prone environments (de la Fuente Cantó et al., 2022). In the search of candidate genes for *Pyricularia* QTL on LG5, we identified a gene Pgl_GLEAN_10037626 that encodes for tetratricopeptide repeat (TPR) domains that are structural motifs comprising tandem repeats of 34 amino acids that facilitate protein-protein interactions (Blatch and Lässle, 1999). In cereals, TPR-containing proteins play significant roles in various biological processes, including disease resistance (Schapire et al., 2006). For instance, in rice, the TPR-domain RNA-binding protein BSR-K1 has been identified as a negative regulator of broad-spectrum disease resistance. A loss-of-function mutation in the *Bsr-k1* gene results in enhanced resistance to multiple pathogens, including *Magnaporthe oryzae* (anamorph: *Pyricularia oryzae*) and *Xanthomonas oryzae* pv. *oryzae* (Zhou et al., 2018). This mutation leads to the production of a truncated BSR-K1 protein that cannot bind to the mRNAs of phenylalanine ammonia-lyase (PAL) genes, resulting in increased accumulation of PAL transcripts. The elevated PAL levels enhance the biosynthesis of secondary metabolites like lignin, thereby strengthening the plant’s defense mechanisms without adversely affecting yield (Zhou et al., 2018). Additionally, another TPR-domain protein in rice, OsTPR1, has been shown to bind with a chitinase from *Magnaporthe oryzae* thus allowing the plant to exhibit an immune response (Yang et al., 2019). Similarly in wheat, TPR-containing proteins have been implicated in regulating agronomic traits that may influence disease resistance. For instance, the TPR protein TaTPR-B1 has been shown to regulate spike compactness, a trait that can affect susceptibility to certain pathogens (Zhu et al., 2025). While the direct role of TaTPR-B1 in disease resistance requires further investigation, its influence on spike architecture suggests a potential indirect effect on pathogen interactions. Hence, the gene may be responsible for *Pyricularia* leaf spot resistance in pearl millet.

*Meloidogyne incognita* are plant-parasitic nematodes that cause significant damage to pearl millet in the southern states where it is grown. Additionally, their presence in the soil affects rotational crops, especially peanuts (*Arachis hypogaea* L.) and cotton (*Gossypium hirsutum* L.). We identified major QTLs for resistance to root-knot nematode (RKN) egg masses on LG2 between markers S2_8057 and S2_8333. Previously, a major QTL for resistance to southern root-knot nematode, QMi-LG2, with a LOD score of 14, explained 32.0% of the phenotypic variance was also mapped to LG2 using an AFLP and SSR-based genetic map (Liu, 2012).

Interestingly, in this study using the same phenotyping data, we report three major QTLs on LG2 with high LOD scores (>8), explaining 32.51%, 28.37%, and 23.84% of the phenotypic variance, indicating a strong association of root-knot nematode egg masses on LG2. The heritability values for nematode and leaf spot resistance were also high. For nematode resistance, the RKN resistance gene from Tift 454 is incompletely dominant or semidominant. The parent Tift 454 is almost immune, the F_1_ generation is resistant but still has egg masses, and the parent Tift 99B is susceptible. The ancestors of Tift 454 include Tift 23D_2_A_1_ and napiergrass (*Pennisetum purpureum* Schumacher) (Hanna et al., 2005a). Previous studies have suggested that the nematode resistance gene may have originated from napiergrass (Liu, 2012). Among 408 unique genes identified in the egg mass QTL region, the gene Pgl_GLEAN_10018312, encodes for Thaumatin-like proteins (TLPs), the members of the pathogenesis-related (PR) protein family, specifically classified under PR-5. They are recognized for their role in plant defense mechanisms against various pathogens, including fungi and bacteria (Sharma et al., 2022). Nevertheless, the gene Pgl_GLEAN_10018133 encodes for plant disease resistance response protein may be implicated for nematode resistance.

Initial scanning for total number of genes within QTL marker intervals yielded 19,762 genes within the QTL regions. Further refining and annotation gave us around 10,705 annotated genes within these 45 QTL regions, including those coding for heat shock protein Hsp90 (**Supplementary Tables S6**, **S7**). Previously, 28 Hsp20 genes were identified based on transcriptome profiling of pearl millet under high-temperature stress (Mukesh Sankar et al., 2021). Hsp90 plays crucial roles in protein folding, stress response, and developmental processes, which are vital for coping with heat and drought stress, making it a target for improving crop resilience.

In summary, next-generation sequencing (NGS) technologies play a prominent role in improving linkage map saturation and its utility in breeding programs. The major QTLs identified in this study and the genes reported in these QTL regions can be used in genomics-assisted breeding to enhance key agronomic and yield-related traits in pearl millet.

## Data Availability

The datasets presented in this study can be found in online repositories. The names of the repository/repositories and accession number(s) can be found in the article/**Supplementary Material**.

## Supplementary material

Supplementary Figure 1 Linkage map along with identified QTL regions.

## References

[B1] Agarwal P. Singh B. R. Gajbe U. Kalambe M. A. Bankar M. (2023). Managing diabetes mellitus with millets: A new solution. Cureus 15, e44908. doi: 10.7759/cureus.44908 37814770 PMC10560538

[B2] Ambawat S. Senthilvel S. Hash C. T. Nepolean T. Rajaram V. Eshwar K.. (2016). QTL mapping of pearl millet rust resistance using an integrated DArT-and SSR-based linkage map. Euphytica 209, 461–476. doi: 10.1007/s10681-016-1671-9

[B3] Andrews D. J. Kumar K. A. (1992). Pearl millet for food, feed, and forage. Adv. Agronomy 48, 89–139. doi: 10.1016/S0065-2113(08)60936-0

[B4] Azhaguvel P. (2001). Linkage map construction and identification of QTLs for downy mildew (*Sclerospora graminicola*) resistance in pearl millet [*Pennisetum glaucum* (L.) R. Br.], Ph.D. Thesis. TNAU, Coimbatore.

[B5] Bernardo R. (2013). Genome-wide markers for controlling background variation in association mapping. Plant Genome 6, 1–9. doi: 10.3835/plantgenome2012.11.0028

[B6] Bertin I. Zhu J. H. Gale M. D. (2005). SSCP-SNP in pearl millet—a new marker system for comparative genetics. Theor. Appl. Genet. 110, 467–1472. doi: 10.1007/s00122-005-1981-0 15809850

[B7] Bidinger F. R. T. Nepolean C. T. Hash R. S. Yadav Howarth C. J. (2007). Quantitative trait loci for grain yield in pearl millet under variable post flowering moisture conditions. Crop Sci. 47, 969–980. doi: 10.2135/cropsci2006.07.0465

[B8] Blatch G. L. Lässle M. (1999). The tetratricopeptide repeat: a structural motif mediating protein-protein interactions. BioEssays 21, 932–939. doi: 10.1002/(SICI)1521-1878(199911)21:11<932::AID-BIES5>3.0.CO;2-N 10517866

[B9] Burgarella C. Cubry P. Kane N. A. Varshney R. K. Mariac C. Liu X.. (2018). A Western Sahara centre of domestication inferred from pearl millet genomes. Nat. Ecol. Evol. 2, 1377–1380. doi: 10.1038/s41559-018-0643-y 30082736

[B10] Chakraborty A. Viswanath A. Malipatil R. Semalaiyappan J. Shah P. Ronanki S.. (2022). Identification of candidate genes regulating drought tolerance in pearl millet. Int. J. Mol. Sci. 23, 6907. doi: 10.3390/ijms23136907 35805919 PMC9266394

[B11] Chelpuri D. Sharma R. Durga K. K. Katiyar P. Mahendrakar M. D. Singh R. B.. (2019). Mapping quantitative trait loci (QTLs) associated with resistance to major pathotype-isolates of pearl millet downy mildew pathogen. Eur. J. Plant Pathol. 154 (4), 983–994. doi: 10.1007/s10658-019-01718-xhemisquy

[B12] Davis A. J. Dale N. M. Ferreira F. J. (2003). Pearl millet as an alternative feed ingredient in broiler diets. J. Appl. Poultry Res. 12, 137–144. doi: 10.1093/japr/12.2.137

[B13] de la Fuente Cantó C. Diouf M. N. Ndour P. M. S. Debieu M. Grondin A. Passot S.. (2022). Genetic control of rhizosheath formation in pearl millet. Sci. Rep. 12 (1), 9205. doi: 10.1038/s41598-022-13234-w 35655088 PMC9163325

[B14] FAOSTAT (2020). FAOSTAT Online Database (Rome, Italy: FAO).

[B15] Faye A. Barnaud A. Kane N. A. Cubry P. Mariac C. Burgarella C.. (2022). Genomic footprints of selection in early-and late flowering pearl millet landraces. Front. Plant Sci. 13. doi: 10.3389/fpls.2022.88063 PMC9597309 36311100

[B16] Gangashetty P. I. Motagi B. N. Pavan R. Roodagi M. B. (2016). Breeding crop plants for improved human nutrition through biofortification: progress and prospects. Adv. Plant Breed. Strategies: Agronom. Abiotic Biotic. Stress Traits pp. 35-76.

[B17] Gupta S. Rangari S. K. Sahu A. (2024). Meta-QTL analysis reveals the important genomics regions for biotic stresses, nutritional quality and yield related traits in pearl millet. CABI Agric. Biosci. 5 (1), p. 36. doi: 10.1186/s43170-024-00230-5

[B18] Hancock D. Kichler J. Smith B. Hicks R. (2018). Georgia forages: grass species. (Georgia: University of Georgia Cooperative Extension) 1351.

[B19] Hanna W. W. Dove R. Hill G. M. Smith R. (1991). “Pearl millet as an animal feed in the US,” in American Society of Agronomy Abstracts, Southern Branch(Fort Worth, Texas).

[B20] Hanna W. W. Wells H. D. (1989). Inheritance of *Pyricularia* leaf spot resistance in pearl millet. J. Hered. 80, 145–147. doi: 10.1093/oxfordjournals.jhered.a110814

[B21] Hanna W. Wilson J. Timper P. (2005a). Registration of pearl millet parental line Tift 454. Crop Sci. 45, 2670–2672. doi: 10.2135/cropsci2005.0171

[B22] Hanna W. Wilson J. Timper P. (2005b). Registration of pearl millet parental lines Tift 99 [D. sub. 2][A. sub. 1]/[B. sub. 1. Crop Sci. 45, 2671–2672. doi: 10.2135/cropsci2005.0172

[B23] Holbrook C. C. Knauft D. A. Dickson D. W. (1983). A technique for screening peanut for resistance to Meloidogyne arenaria. Plant Disease 67, 957–958. doi: 10.1094/PD-67-957

[B24] Kannan B. Senapathy S. Bhasker Raj A. G. Chandra S. Muthiah A. Dhanapal A. P.. (2014). Association analysis of SSR markers with phenology, grain, and stover-yield related traits in pearl millet (*Pennisetum glaucum* (L.) R. Br.). Sci. World J. 2014, 562327. doi: 10.1155/2014/562327 PMC3910278 24526909

[B25] Kumar S. Hash C. T. Nepolean T. Satyavathi C. T. Singh G. Mahendrakar M. D.. (2017). Mapping QTLs controlling flowering time and important agronomic traits in pearl millet. Front. Plant Sci. 8. doi: 10.3389/fpls.2017.01731 PMC5742331 29326729

[B26] Kumar S. Hash C. T. Singh G. Nepolean T. Srivastava R. (2021). Mapping QTLs for important agronomic traits in an Iniadi-derived immortal population of pearl millet. Biotechnology Notes. 2, 26–32. doi: 10.1016/j.biotno.2021.06.001

[B27] Liu L. (2012). Genetic mapping and quantitative trait locus (QTL) analysis of root-knot nematode resistance in pearl millet. Ph.D. dissertation Vol. 3 (Athens, GA, USA: University of Georgia).

[B28] Moin M. Bommineni P. R. Tyagi W. (2024). Exploration of the pearl millet phospholipase gene family to identify potential candidates for grain quality traits. BMC Genomics 25, 581. doi: 10.1186/s12864-024-10504-x 38858648 PMC11165789

[B29] Morgan R. N. Wilson J. P. Hanna W. W. (1998). Molecular markers for rust and *Pyricularia* leaf spot disease resistance in pearl millet. Theor. Appl. Genet. 96, 413–420. doi: 10.1007/s001220050757 24710880

[B30] Moumouni K. H. Kountche B. A. Jean M. Hash C. T. Vigouroux Y. Haussmann B. I. G.. (2015). Construction of a genetic map for pearl millet, *Pennisetum glaucum* (L.) R. Br., using a genotyping-by-sequencing (GBS) approach. Mol. Breeding 35, 5.

[B31] Mukesh Sankar S. Tara Satyavathi C. Barthakur S. Singh S. P. Bharadwaj C. Soumya S. L. (2021). Differential modulation of heat-inducible genes across diverse genotypes and molecular cloning of a sHSP from pearl millet [*Pennisetum glaucum* (L.) R. Br. Front. Plant Sci. 12. doi: 10.3389/fpls.2021.659893 PMC8324246 34335644

[B32] Parvathaneni R. K. Jakkula V. Padi F. K. Faure S. Nagarajappa N. Pontaroli A. C.. (2013). Fine-mapping and identification of a candidate gene underlying the *d2* dwarfing phenotype in pearl millet, *Cenchrus americanus* (L.) Morrone. G3: Genes Genomes Genet. 3, 563–572. doi: 10.1534/g3.113.005587 PMC3583462 23450459

[B33] Passot S. Gnacko F. Moukouanga D. Lucas M. Guyomarc’h S. Ortega B. M.. (2016). Characterization of pearl millet root architecture and anatomy reveals three types of lateral roots. Front. Plant Sci. 7, 829. doi: 10.3389/fpls.2016.00829 27379124 PMC4904005

[B34] Pei J. Umapathy V. R. Vengadassalapathy S. Hussain S. F. J. Rajagopal P. Jayaraman S.. (2022). A review of the potential consequences of pearl millet (*Pennisetum glaucum*) for diabetes mellitus and other biomedical applications. Nutrients 14, 2932. doi: 10.3390/nu14142932 35889889 PMC9322144

[B35] Poncet V. Lamy F. Devos K. M. Gale M. D. Sarr A. Robert T. (2000). Genetic control of domestication traits in pearl millet (*Pennisetum glaucum* L., Poaceae). Theor. Appl. Genet. 100, 147–159. doi: 10.1007/s001220050020 12582601

[B36] Pucher A. Hash C. T. Wallace J. G. Han S. Leiser W. L. Haussmann B. I. (2018). Mapping a male-fertility restoration locus for the A 4 cytoplasmic-genic male-sterility system in pearl millet using a genotyping-by-sequencing-based linkage map. BMC Plant Biol. 18, 1–11. doi: 10.1186/s12870-018-1267-8 29665794 PMC5905146

[B37] Punnuri S. Wallace J. Knoll J. (2024). “High-density genotyping for pearl millet linkage map improvement with next-generation sequencing data,” in The Pearl Millet Genome (Springer International Publishing, Cham), 97–105.

[B38] Punnuri S. M. Wallace J. G. Knoll J. E. Hyma K. E. Mitchell S. E. Buckler E. S.. (2016). Development of a high-density linkage map and tagging leaf spot resistance in pearl millet using genotyping-by-sequencing markers. Plant Genome 9 (2). doi: 10.3835/plantgenome2015.10.0106 27898821

[B39] Rai K. N. Gowda C. L. L. Reddy B. V. S. Sehgal S. (2008). Adaptation and potential uses of sorghum and pearl millet in alternative and health foods. Compr. Rev. Food Sci. Food Saf. 7, 320–396. doi: 10.1111/j.1541-4337.2008.00049.x 33467790

[B40] Rajaram V. Nepolean T. Senthilvel S. Varshney R. K. Vadez V. Srivastava R. K.. (2013). Pearl millet [*Pennisetum glaucum* (L.) R. Br.] consensus linkage map constructed using four RIL mapping populations and newly developed EST-SSRs. BMC Genomics 14, 1–16. doi: 10.1186/1471-2164-14-159 23497368 PMC3606598

[B41] Satyavathi C. T. Ambawat S. Khandelwal V. Srivastava R. K. (2021). Pearl millet: A climate-resilient nutricereal for mitigating hidden hunger and provide nutritional security. Front. Plant Sci. 12. doi: 10.3389/fpls.2021.659938 PMC8475763 34589092

[B42] Schapire A. L. Valpuesta V. Botella M. A. (2006). TPR proteins in plant hormone signaling. Plant Signal Behav. 1, 229–230. doi: 10.4161/psb.1.5.3491 19704665 PMC2634123

[B43] Sehgal D. Rajaram V. Armstead I. P. Vadez V. Yadav Y. P. Hash C. T.. (2012). Integration of gene-based markers in a pearl millet genetic map for identification of candidate genes underlying drought tolerance quantitative trait loci. BMC Plant Biol. 12, 9. doi: 10.1186/1471-2229-12-9 22251627 PMC3287966

[B44] Sharma P. C. Sehgal D. Singh D. Singh G. Yadav R. S. (2011). A major terminal drought tolerance QTL of pearl millet is also associated with reduced salt uptake and enhanced growth under salt stress. Mol. Breeding 27, 207–222. doi: 10.1007/s11032-010-9423-3

[B45] Sharma A. Sharma H. Rajput R. Pandey A. Upadhyay S. K. (2022). Molecular characterization revealed the role of thaumatin-like proteins of bread wheat in stress response. Front. Plant Sci. 12. doi: 10.3389/fpls.2021.807448 PMC8786798 35087559

[B46] Singh S. Prakash G. Nanjundappa S. Malipatil R. Kalita P. Satyavathi T. C.. (2024a). Novel SNPs linked to blast resistance genes identified in pearl millet through genome-wide association models. Int. J. Mol. Sci. 25, 12048. doi: 10.3390/ijms252212048 39596115 PMC11593765

[B47] Singh S. Yadav C. B. Lubanga N. Hegarty M. Yadav R. S. (2024b). Genome-wide SNPs and candidate genes underlying the genetic variations for protein and amino acids in pearl millet (*Pennisetum glaucum*) germplasm. Planta 260, 63. doi: 10.1007/s00425-024-04495-y 39068266 PMC11283402

[B48] Srivastava R. K. Bollam S. Pujarula V. Pusuluri M. Singh R. B. Potupureddi. G.. (2020). Exploitation of heterosis in pearl millet: a review. Plants 9, 807. doi: 10.3390/plants9070807 32605134 PMC7412370

[B49] Subbulakshmi K. Karthikeyan A. Murukarthick J. Dhasarathan M. Naveen R. Sathya M.. (2024). Consensus genetic linkage map and QTL mapping allow to capture the genomic regions associated with agronomic traits in pearl millet. Planta 260, 57. doi: 10.1007/s00425-024-04487-y 39039303

[B50] Thakur R. P. Sharma R. Rao V. P. (2011). Screening Techniques for Pearl Millet Diseases. Technical Report Vol. 1. Andhra Pradesh, India, International Crops Research Institute for the Semi-Arid Tropics.

[B51] Thudi M. Palakurthi R. Schnable J. C. Chitikineni A. Dreisigacker S. Mace E.. (2021). Genomic resources in plant breeding for sustainable agriculture. J. Plant Physiol. 257, 153351. doi: 10.1016/j.jplph.2020.153351 33412425 PMC7903322

[B52] Timper P. Wilson J. P. (2006). Root-knot nematode resistance in pearl millet from West and East Africa. Plant Dis. 90, 339–344. doi: 10.1094/PD-90-0339 30786559

[B53] Timper P. Wilson J. P. Johnson A. W. Hanna W. W. (2002). Evaluation of pearl millet grain hybrids for resistance to *Meloidogyne* spp. and leaf blight caused by *Pyricularia grisea* . Plant Dis. 86, 909–914. doi: 10.1094/PDIS.2002.86.8.909 30818647

[B54] Tomar M. Bhardwaj R. Kumar M. Singh S. P. Krishnan V. Kansal R.. (2021). Nutritional composition patterns and application of multivariate analysis to evaluate indigenous Pearl millet (*Pennisetum glaucum* (L.) R. Br.) germplasm. J. Food Composition Anal. 103, 104086. doi: 10.1016/j.jfca.2021.104086

[B55] Varshney R. K. Shi C. Thudi M. Mariac C. Wallace J. Zhang H.. (2017). Pearl millet genome sequence provides a resource to improve agronomic traits in arid environments. Nat. Biotechnol. 35, 1–13. doi: 10.1038/nbt.3943 PMC6871012 28922347

[B56] Varshney R. K. Thudi M. Pandey M. K. Tardieu F. Ojiewo C. Vadez V.. (2018). Accelerating genetic gains in legumes for the development of prosperous smallholder agriculture: integrating genomics, phenotyping, systems modelling and agronomy. J. Exp. Bot. 69, 3293–3312. doi: 10.1093/jxb/ery088 29514298

[B57] Vengadessan V. Rai K. N. Kannan Bapu J. R. Hash C. T. Bhattacharjee R. Senthilvel S.. (2013). Construction of genetic linkage map and QTL analysis of sink-size traits in pearl millet (*Pennisetum glaucum*). Int. Scholarly Res. Notices 2013 (1), p.471632. doi: 10.5402/2013/471632

[B58] Voorrips R. E. (2002). MapChart: Software for the graphical presentation of linkage maps and QTLs. J. Hered. 93, 77–78. doi: 10.1093/jhered/93.1.77 12011185

[B59] Wang S. Basten C. J. Zeng Z. B. (2012). Windows QTL Cartographer 2.5 (Raleigh, North Carolina, USA: Department of Statistics, North Carolina State University).

[B60] Wilson J. P. Hanna W. W. (1992). Effects of gene and cytoplasm substitutions in pearl millet on leaf blight epidemics and infection by *Pyricularia grisea* . Phytopathology 82, 839–842. doi: 10.1094/Phyto-82-839

[B61] Yadav O. P. Gupta S. K. Govindaraj M. Sharma R. Varshney R. K. Srivastava R. K.. (2021). Genetic gains in pearl millet in India: Insights into historic breeding strategies and future perspective. Front. Plant Sci. 12. doi: 10.3389/fpls.2021.645038 PMC8042313 33859663

[B62] Yadav R. S. Hash C. T. Bidinger F. R. Cavan G. P. Howarth C. J. (2002). Quantitative trait loci associated with traits determining grain and stover yield in pearl millet under terminal drought-stress conditions. Theor. Appl. Genet. 104, 67–83. doi: 10.1007/s001220200008 12579430

[B63] Yadav R. S. Hash C. T. Bidinger F. R. Devos K. M. Howarth C. J. (2004). Genomic regions associated with grain yield and aspects of post flowering drought tolerance in pearl millet across stress environments and tester background. Euphytica 136, 265–277. doi: 10.1023/b:euph.0000032711.34599.3a

[B64] Yang C. Yu Y. Huang J. Meng F. Pang J. Zhao Q.. (2019). Binding of the *Magnaporthe oryzae* chitinase MoChia1 by a rice tetratricopeptide repeat protein allows free chitin to trigger immune responses. Plant Cell. 31, 172–188. doi: 10.1105/tpc.18.00382 30610168 PMC6391695

[B65] Zhou X. Liao H. Chern M. Yin J. Chen Y. Wang J.. (2018). Loss of function of a rice TPR-domain RNA-binding protein confers broad-spectrum disease resistance. Proc. Natl. Acad. Sci. 115, 3174–3179. doi: 10.1073/pnas.1705927115 29432165 PMC5866533

[B66] Zhu J. Huang F. Zhai H. Zheng Y. Yu J. Chen Z.. (2025). The Tetratricopeptide repeat protein TaTPR-B1 regulates spike compactness in bread wheat. Plant Physiol. 197, kiae546. doi: 10.1093/plphys/kiae546 39405430

