# Supplementary material for: Mapping QTLs for Pyricularia leaf spot, nematode resistance, and yield related traits in pearl millet [Cenchrus americanus (L.) Morrone]
Source: Front Plant Sci. 2025 Jun 30;16:1588485. doi: 10.3389/fpls.2025.1588485 (PMC12256767; doi:10.3389/fpls.2025.1588485)
Supplement: Supplementary Figure 1 — Linkage map along with identified QTL regions. [file Presentation1.pptx]

## Slide 1
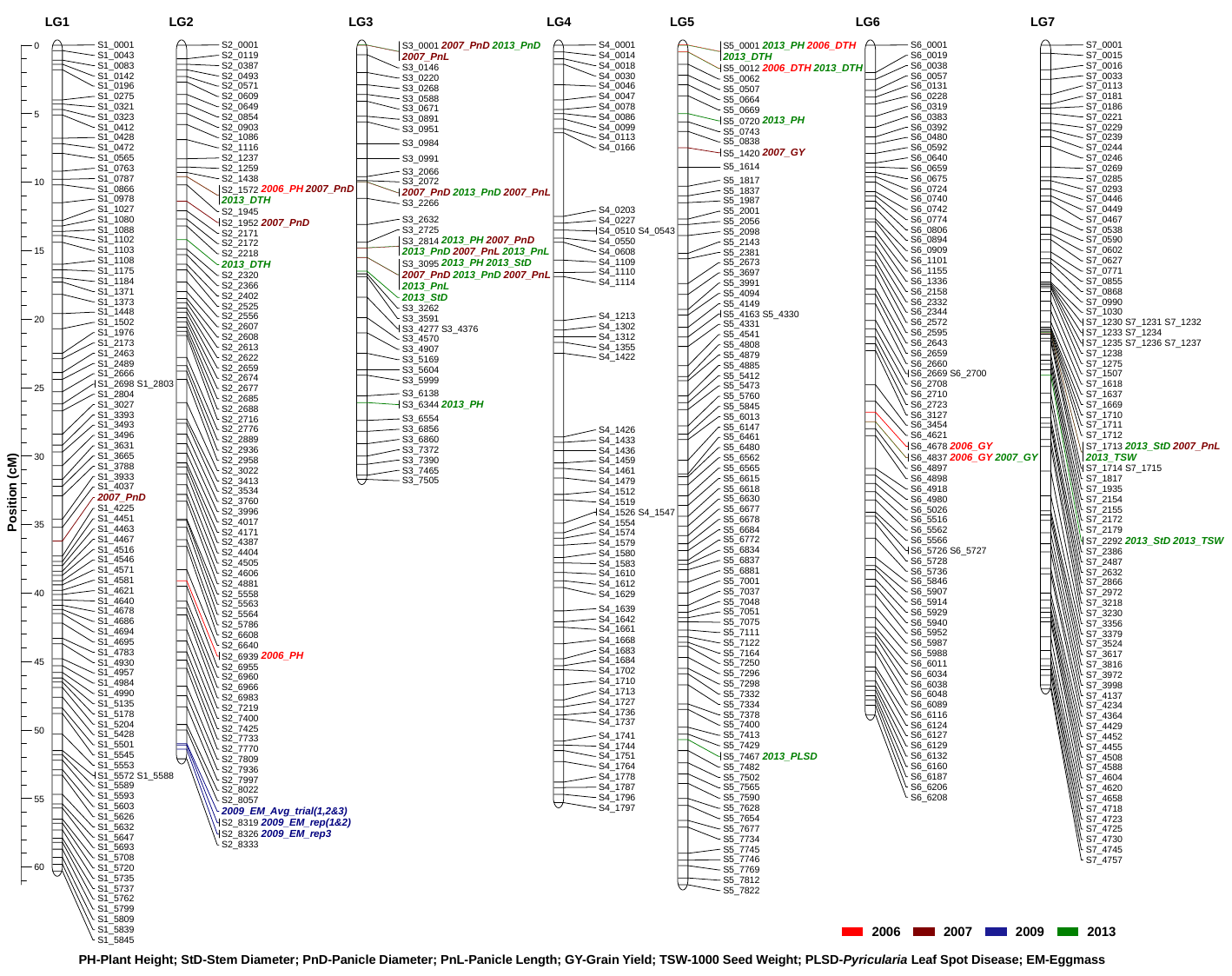

LG2
LG3
LG4
LG5
LG6
LG7
LG1
Position (cM)
2006
2007
2009
2013
PH-Plant Height; StD-Stem Diameter; PnD-Panicle Diameter; PnL-Panicle Length; GY-Grain Yield; TSW-1000 Seed Weight; PLSD-Pyricularia Leaf Spot Disease; EM-Eggmass
